# Morphoelastic modelling of pattern development in the petal epidermal cell cuticle

**DOI:** 10.1098/rsif.2023.0001

**Published:** 2023-07-05

**Authors:** Carlos A. Lugo, Chiara Airoldi, Chao Chen, Alfred J. Crosby, Beverley J. Glover

**Affiliations:** ^1^ Department of Plant Sciences, University of Cambridge, Downing Street, Cambridge CB2 3EA, UK; ^2^ Sainsbury Laboratory, University of Cambridge, Cambridge CB2 1LR, UK; ^3^ Polymer Science and Engineering Department, University of Massachusetts Amherst, Amherst, MA 01003, USA

**Keywords:** flower, wrinkle, mechanics

## Abstract

We use the model system *Hibiscus trionum* as a vehicle to study the origin and propagation of surface nano-ridges in plant petal epidermal cells by tracking the development of the cell shape and the cuticle. In this system, the cuticle develops two distinct sub-layers, (i) an uppermost layer which increases in thickness and in-plane extension and (ii) a substrate, composed of cuticular and cell wall material. We quantify the pattern formation and geometrical changes and then postulate a mechanical model assuming that the cuticle behaves as a growing bi-layer. The model is a quasi-static morphoelastic system and it is numerically investigated in two- and three-dimensional settings, using different laws of film and substrate expansion and boundary conditions. We recreate several features of the observed developmental trajectories in petals. We establish the respective roles of the layers’ stiffness mismatch, the underlying cell-wall curvature, the cell in-plane expansion and the thickness growth rates of the layers in determining the observed pattern features, such as the variance observed in the amplitude and wavelength of the cuticular striations. Our observations provide evidence which justifies the growing bi-layer description, and provide valuable insights into why some systems develop surface patterns and others do not.

## 1. Introduction

The variety of topographic patterns found on the surface of plant petal and leaf epidermal cells has attracted the attention of researchers from disciplines ranging from developmental biology [1,2] to material sciences [3]. These patterns have been shown to mediate several forms of interaction with the environment. For instance, it has been demonstrated [1,4,5] that such patterned petals can act as optical and tactile cues for pollinators, provide some flowers with structural colour, and may also contribute to altered flower-pollinator adhesion and can regulate hydrophobicity [6].

**Figure 1.**
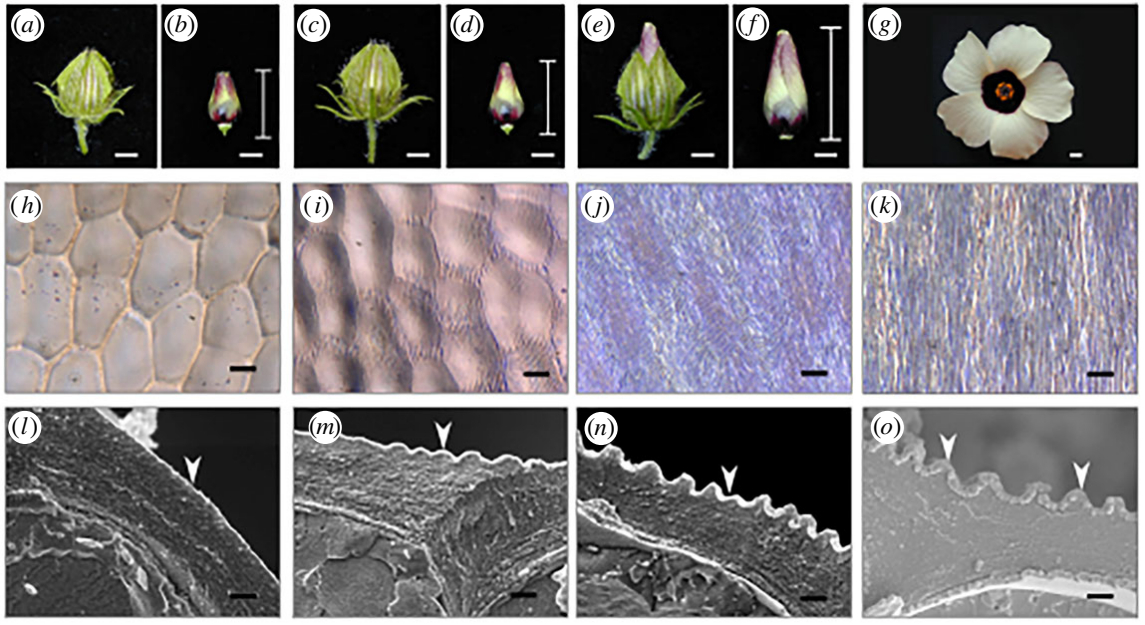
Development of *Hibiscus trionum* flower, epidermal cells and surface morphologies. (*a*–*g*) Pictures of *H. trionum* buds (*a*–*f*) and an open flower (*g*), scale bars 1mm. Petals of the flower buds are covered by an epicalyx that has been removed in *b*,*d*,*f*. The bud size is referred to as the bottom to tip length after the epicalyx has been removed. (*h*,*i*,*j*,*k*) Microscopic images of epidermal cell surfaces in the purple adaxial region of the petal at the stage shown above (scale bar 10 μm). (*l*,*m*,*n*,*o*) CryoSEM-fractures of *H. trionum* petal epidermal cells at the stage shown above (scale bars 1 μm). Panels (*a*,*b*,*h*,*l*) are pictures of a bud during the pre-striation stage (10 mm bud, measured as specified in the Material and methods, early stage 3 with reference to [7]); *c*,*d*,*i*,*m* at the onset of striation (12.5–13 mm bud late stage 3 in [7]); *e*,*f*,*j*,*n* are from a mature bud 1 day before flower opening (19–20 mm bud, stage 4 in [7]). *g*,*k*,*o* pictures are from an open flower (stage 5 in [7]). The arrows in (*l*–*o*) indicate the development of the cuticle film.

A striking example of pattern functionality is provided by the model system *Hibiscus trionum* (figure 1) in which the pattern consists of quasi-periodic nano ridges acting as a light diffracting grating array, conferring structural colour to the flower. The pattern forms on one of the two sub-domains of the petal’s adaxial surface which are defined by their epidermal cell shapes and which roughly coincide with the white and pigmented regions. The white domain consists of conical-shaped cells that do not develop surface pattern. The rest of the petal is composed of cells with an elongated in-plane rectangular shape located mostly in the purple pigmented region of the tissue and where the cell surface develops a distinctive striated pattern.

The striated pattern aligns parallel to the major axis of the cells and forms on the cuticle, which is the outermost layer of the epidermis. Plant cuticles are extracellular polymer layers that act as a physical defence against a number of environmental agents. They comprise two chemically distinguishable sub-layers: a base layer that starts at the cell wall, which we refer to as the ‘cuticular substrate’, and an upper surface-most layer or ‘cuticle film’ [8–10]. Such bi-layered structures play a significant role in systems that develop periodic surface deformations, as a result of a compression-induced elastic instability. In the case of *H. trionum*, it has been shown that the cuticle behaves like a bi-layer composite, as the patterns can be induced by uniaxial stretching of the cuticles before the pattern occurs naturally [11]. The striated domain generates an iridescent reflectance spectrum which as reported in [3] is also different from that of a perfectly flat periodic grating and has served as inspiration in the design of new materials.

The origin of the surface deformation has been hypothesized to be mechanical in nature, a hypothesis that has also been applied to other systems of tissue-wide cuticle patterning [2], which may be ordered, disordered, overlapping or apparently random. Specifically, it may be due to a mechanical instability associated with cuticle overproduction. In [12], the authors assume the cuticle to be a homogeneous incompressible, mechanically isotropic layer, and propose that cuticle production is described by an adjustable parameter, which turns the in-plane stress components into tensile or compressive as the cuticle is anisotropically stretched due to cellular in-plane growth. This purely elastic formulation, however, does not address issues such as the existence of buckled configurations associated with the stress tensors proposed, the irreversible nature of the patterns or the critical nature of the elastic instability, i.e. the existence of in-plane stress threshold values which need to be exceeded for the material to buckle. Nor does it provide any understanding of pattern features such as the amplitude or wavelength of striations and their relation to the material properties of the cuticle. A more comprehensive description of the mechanics behind patterned cuticles in petals is given in [13] by considering the cuticle as a bi-layered composite described by linear stress–strain constitutive relations and linking stress to in-plane cell and petal shape geometrical changes, which then determine the pattern orientation but without explicitly addressing the developmental aspects we discuss here.

A bilayer composite develops a surface periodic pattern or wrinkling/buckling instability in the upper surface if an applied in-plane stress in a given direction exceeds a threshold value [14,15]. The resulting pattern is oriented in the perpendicular direction to the applied in-plane stress [16–20]. The critical threshold and pattern features depend on the elastic properties of the composite, its geometry and boundary conditions. For rectangular flat volumes, these dependencies are fairly general, regardless of the material model specifics, provided the elastic responses are isotropic [16,18,20], and are given by the scaling rules at the onset of the instability, namely, the wavelength

1.1 $$\lambda_{c}=\gamma_{1}hR^{1/3}\;\;$$

and the critical strain

1.2 $$\varepsilon_{c}=\gamma_{2}R^{-(2/3)},$$

where *h* is the film thickness, *R* the ratio of the elastic response modulus of the film and substrate, and *γ*_1_ and *γ*_2_ are constants. Equation (1.1) states that a thicker upper film induces a larger wavelength, whereas equation (1.2) means that larger elastic mismatch between the film/substrate stiffness results in lower buckling thresholds. As described elsewhere [18,21], these patterns emerge as trade-off states between the energetically favourable states of the slender film and the substrate under the same stress.

The universality of the buckling mechanism [14,22,23] means that the trends in equations (1.1) and (1.2) are followed by a large class of material models, regardless of the origin of the in-plane stress. For instance, periodic buckling patterns can be achieved by several settings involving uniaxial compression or unidirectional in-plane growth of the layers [24]. If in-plane stress acts on more than one direction, the possible instabilities which can be triggered range from chequerboards and hexagonal arrays to herringbones [20,21].

In this work, we present results of a detailed developmental analysis of the cell geometry and pattern features in the *H. trionum* petal, which are then used to formulate morphomechanical models of the system [25]. As discussed below, this results in configurations of an irreversible nature and with deformations which are beyond the linear theory. Investigation of the models allowed us to elucidate the interplay of cuticle production, cell elongation and cell bulging in shaping the pattern and also permit us to pinpoint the locations where the pattern is triggered. Specifically, we explore the effects that different growth rates have on both reaching the buckling threshold and on the pattern features. Our results explain why some plant cuticles form surface patterns whereas others do not and clarify how this is related to cuticle synthesis and cuticle structural and mechanical properties [26]. Our results are also relevant in the development of bioinspired engineered materials where some degree of tunability is a desired feature [3].

## 2. Results

From a petal development perspective, it is not intuitive to understand the mechanism behind the source of stress which may induce a buckling instability. As we show next, quantitative measurements of changes of the cell geometry support the case for in-plane compressive stress induced by a larger expansion of the cuticle film in comparison with that of the substrate and underlying cells, in the direction orthogonal to the pattern ridges, to be the mechanism responsible for the origin of the buckling instability in *H. trionum*.

### 2.1. Description of hibiscus flower epidermis and its development

In *H. trionum*, the pattern occurs in the uppermost layer on the external face of the epidermal cells, i.e. in the plant cuticle. The pattern develops from a flat surface in the early stages of petal development (figure 1*a*,*b*,*h*,*l*). During the onset-stage striations appear first around the cell edges (figure 1*c*,*d*,*i*,*m*). We distinguish these two stages, which were both encompassed by stage 3 in the developmental series described previously [7]. The ridges populate the entire cell surface at the mature bud stage, previously described as stage 4 [7] (figure 1 *e*,*f*,*j*,*n*), assuming their final semi-ordered configuration in the open flower (stage 5 [7] figure 1*g*,*k*,*o*).

**Figure 2.**
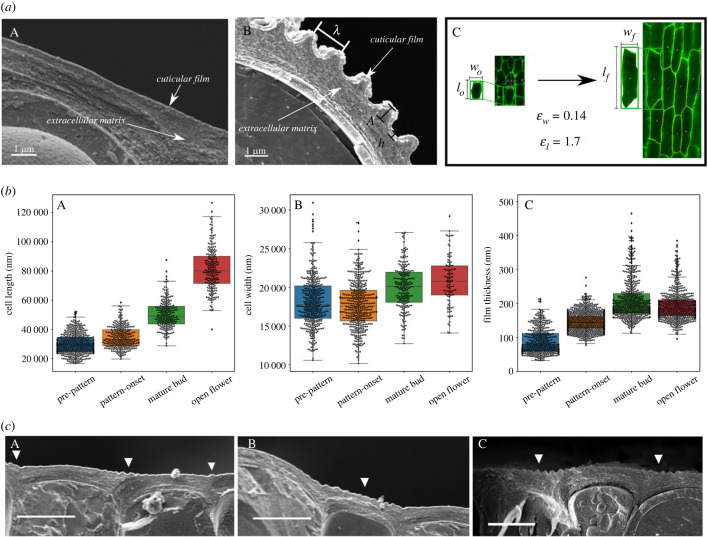
In-plane geometrical and morphological surface changes during development of *H. trionum* petal epidermal cells. (*a*) The cuticle film thickens and the cells elongate as the petal develops. (*b*) Box plots of the change in cell length (A), cell width (B) and (C) cuticle film thickness. (*c*) The pattern starts forming on flatter regions before developing across the entire domain which exhibits structural colour. (*a*) CryoSEM cross-sections of a surface cell before and after the pattern is formed and stereo-microscopy images illustrating the width *w* and length *l* in-plane changes for a cell at the onset of pattern formation *w*_*o*_ and *l*_*o*_ (12.0 mm buds) to cells in a fully developed flower *w*_*f*_ and *l*_*f*_ which for the average values give $\varepsilon_{w}=0.14$ and $\varepsilon_{l}=1.7$ as described in the text. (*b*) Results of measuring the in-plane cell length (A), cell width (B) and thickness of the cuticle film (C) as the petal develops. (*c*) Cryo SEM fractures captured at the onset of pattern formation (12.5 –13 mm bud). (A,C) Striations originating between cells, (B) striations originating in areas with less curvature (scale bar 10 μm).

The sequence in figure 1*l*–*o* and additional cryo sections for before and after pattern formation (figure 2*a*,*b*), show that the cuticle film layer is not distinguishable from the rest of the cuticle at an early stage of development, but appears very clearly as a distinct layer in the fully developed phenotype. The fully formed pattern image in figure 2*b* shows features used to characterize the pattern, namely the amplitude *A*, wavelength *λ* and the cuticle film thickness *h*.

Both the in-plane width *w* and length *l* increase, except for a slight decrease in *w* before the striations start to form, where cell division is still more frequent than in-plane expansion. Figure 2*b* shows measurements of these as well as for the thickness of the cuticle film. The changes $\varepsilon_{u}=({\left\langle u\right\rangle }_{f}-{\left\langle u\right\rangle }_{12})/{\left\langle u\right\rangle }_{12}$ of the average in-plane cell width and length〈*u*〉(*u* ∈ {*w*, *l*}) with respect to the average cell widths and lengths for buds of 12 mm 〈*u*〉 _12_ obtained from the data are $\varepsilon_{w}∼0.14$ and $\varepsilon_{l}∼1.7$. Both show a net increase in both directions, most noticeable in the direction which grows parallel to the petal length, as shown in figure 1*l*–*o*. The thickness *h* of the cuticle film increases from a very thin layer to a value which remains almost constant during development. From the start of pattern formation until the flower opens, cell elongation is the main expansion mechanism responsible for the petal surface growth. The in-plane cell expansion and the cuticle thickening indicate that the volume of the cuticle layer is increasing, if such expansion is isotropic, then the cell expansion of the cells in a preferential (length) direction will induce an excess of cuticle in the perpendicular direction (width) effectively compressing the cuticle in that direction.

Figure 2*c* shows several cross-sections taken at the onset of pattern appearance. The pattern starts populating flatter sub-domains of the tissue such as the regions located on top of cell junctions, whereas for regions with larger curvature, pattern initiation is delayed by the bulging of the cell wall due to turgor pressure. This external domain deformation also contributes to a stress distribution as the regions with local positive-signed curvature (concave) are effectively compressed, whereas those with negative-signed curvature (convex) are under tension.

### 2.2. Mechanics

**Figure 3.**
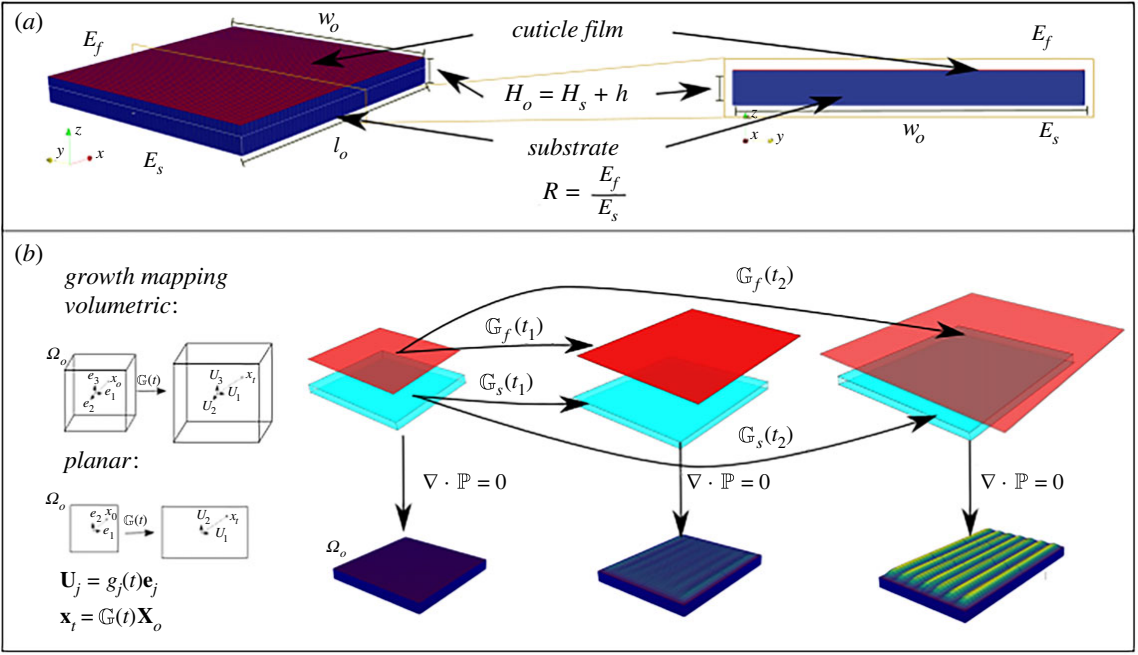
Cuticle bilayer model and the underlying geometry of growth-induced stress. (*a*) The reference configuration $\Omega_{o}$ consists of a volume of initial depth *H*_*o*_ composed of two layers of initial thickess *H*_*s*_ and *h* for the substrate and the cuticular film and the in-plane initial width and lengths *w*_*o*_ and *l*_*o*_. These sub-layers are characterized by their Young’s modulus *E*_*f*_ and *E*_*s*_, which we encode in the parameter *R* = *E*_*f*_/*E*_*s*_. (*b*) The geometry associated with the growth tensors $G_{f}$ and $G_{s}$ which map the reference configuration to ‘grown’ configurations in terms of principal directions. These new configurations need to fulfil the imposed boundary conditions of the elastic problem which, as the figure shows, correspond to calculating the elastic response of accommodating a larger volume into a smaller one which is spanned by the underlying cell in-plane surface.

We consider the cuticle as a bilayer volume consisting of an elastic film resting over a compliant substrate such as that shown in figure 3. The layers are characterized by their elastic properties. Assuming both the cuticle film and substrate cuticular layer to be mechanically isotropic, it is enough to specify the Lamé parameters of each layer to specify a constitutive relation between stress and deformation to model the passive responses.

In this work, we make use of hyperelasticity to describe the cuticles and specify the Lamé numbers in terms of the linear response modulus or Young’s modulus and Poisson’s ratio of each layer. If we consider the cuticle as a near-incompressible material across all the volume, then the latter *ν*_*s*_ = *ν*_*f*_ = *ν* ∼ 0.5 where *f* and *s* stand for film and substrate. The linear response coefficients can be encoded in the stiffness mismatch ratio *R* = *E*_*f*_/*E*_*s*_.

A key difference between synthetic materials and living systems, such as petal surfaces, is that for living matter growth and remodelling are mostly due to internal processes rather than caused by the action of external agents such as an imposed displacement or an external force. For the case under consideration here, we assume that cuticle overproduction translates into a local increase of the volume occupied by every volume element composing the layers. The incorporation of growth into the mechanics framework or morphoelasticity is based on the decomposition of the deformation gradient F as a product of the elastic response A and a growth tensor G,

2.1 F=AG. 

The decomposition equation (2.1) allows us to solve an elastic problem provided we know the underlying growth rates of the solid with respect to a stress-free reference configuration $\Omega_{o}$. The complete formulation of the problem requires us to provide a constitutive equation for the material, boundary conditions and the fulfilment of the relevant balance laws, namely linear momentum, torque and mass. Assuming that the addition of mass into the system is such that the volumetric density remains approximately constant, then the stationary configuration is found by solving the elastic problem

2.2 ∇⋅P=0,

where P is the nominal stress tensor (see Material and methods). The components of the tensor G provide the link between cuticle production and mechanical stress. If *k* refers to the substrate or film, the growth tensors for each sub-layer are

2.3 $$G_{k}=\left(\begin{array}{cc} g_{k,1} & 0\\ 0 & g_{k,2} \end{array}\right),$$

for the dynamics of cross-section only. Whereas for the full volumetric case

2.4 $$G_{k}=\left(\begin{array}{ccc} g_{k,1} & 0 & 0\\ 0 & g_{k,2} & 0\\ 0 & 0 & g_{k,3} \end{array}\right).$$

Any deformation that solves equation (2.2) is a result of cuticle growth and it is irreversible as there are no external forces acting on the volume. The source of compressive stress is therefore provided by the boundary conditions. The cuticle is synthesized locally on the surface of each cell and if the rates of expansion exceed those of cell in-plane elongation in a given direction, then we are trying to accommodate a larger volume within a smaller one, due to the presence of the neighbouring cells (figure 3), in a similar fashion as the expansion of a cake is constrained by the baking container.

### 2.3. Film thickness and turgor bulging

We start by presenting results of planar simulations which emulate the deformations of a cross-section. This is convenient because all of the ingredients responsible for the pattern formation are present at a relatively inexpensive computational effort, allowing us to extend the study to more elaborate settings. The initial stress-free configuration is shown in figure 3 and is given by $\Omega_{o}\;:\;[0,l_{o}]\times [0,H_{o}]$ with *H*_*o*_ = *h* + *H*_*s*_. We choose the values *l*_*o*_ = 2.0, *H_o_* = 0.2, *h*/*H_o_* = 0.008, *R* = 10 and *ν* = 0.45, to mimic the aspect ratios observed in the cryo-fractures shown in figures 1 and 2.

**Figure 4.**
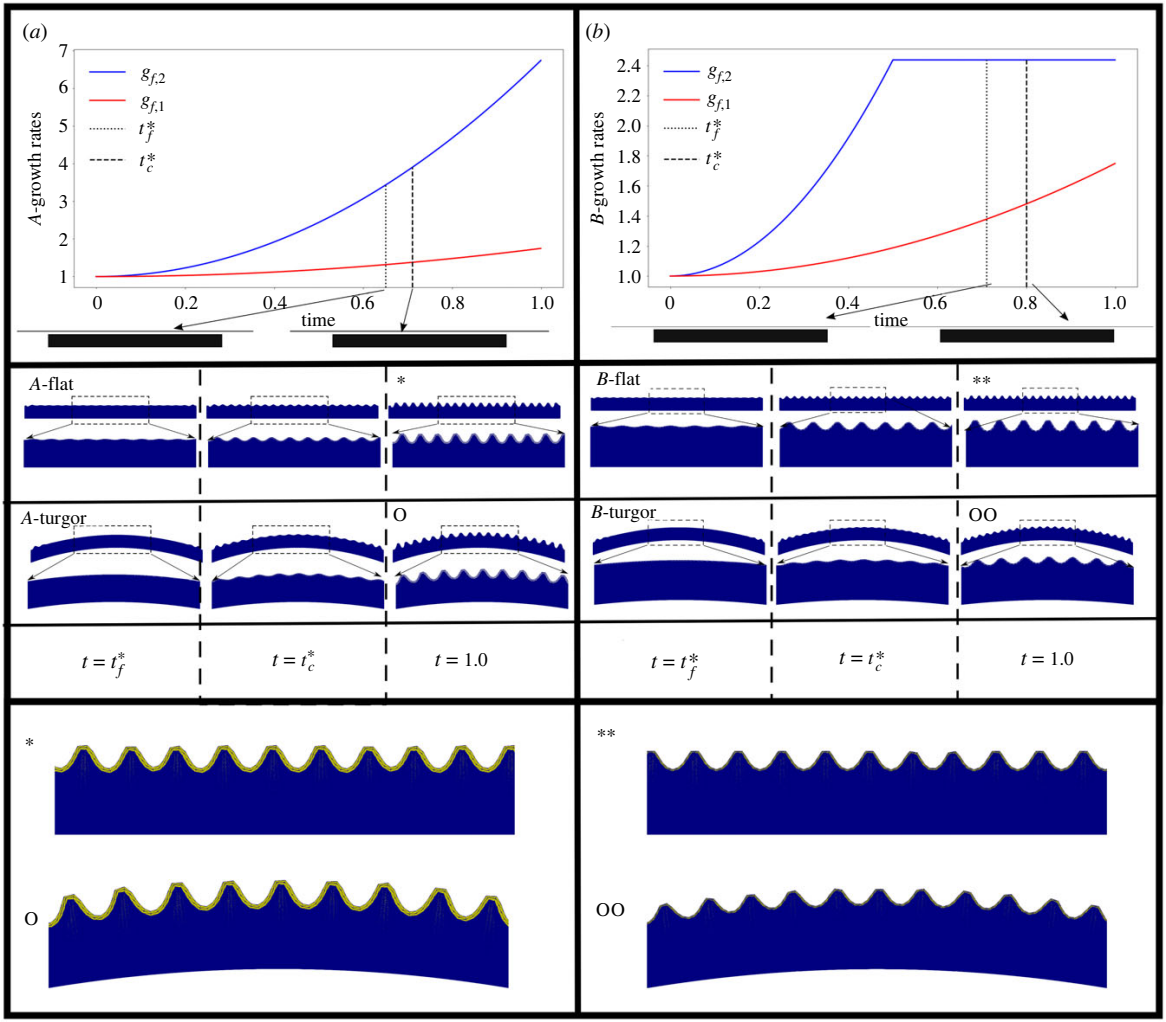
Effect of curved boundaries on triggering the instability. Solutions of the model using two sets of film expansion rates and boundary conditions. Column (*a*) both entries use *g*_*f*,*k*_(*t*) = 1 + (*C*_*k*_/2)*t*^2^, with *C*_1_ = 1.0 and *C*_2_ = 10. Column (*b*) is the same as in (*a*) but with *g*_*f*,2_ stopping at *t* = 0.5. Each case is shown using flat and curved bottom boundaries. The bottom panel of each column shows a close-up of the solutions at *t* = 1.0 labelled by the symbols *, **, o, oo accordingly. For both growth scenarios, the instability threshold is reached sooner using a flat boundary condition than for the curved cases. The reference configuration is the same for all cases, and the bottom boundary is updated in the same way as the growth tensor entries using a function *u*(*x*) = *r*(*t*)*x*(*x* − *w*_*o*_) with *r* = 0.2*t* for *t* < 0.65, this is done to emulate the bulging of cells due to turgor pressure as observed in figures 1 and 2.

In figure 4, we show results of two different sets of growth tensors for the film and for each case two different boundary conditions; the substrate in these cases does not expand. The growth tensors used are shown in the figure. The ones labelled *A* (*a*) are given by *g*_*j*,*f*_(*t*) = 1 + *C*_*j*_*t*^2^, with *C*_1_ = 1 and *C*_2_ = 10*C*_1_ and *t* ∈ [0, 1], for the ones in *B* (*b*) the expansion laws are the same but the thickness stops increasing at *t* = 0.5 before reaching the threshold and while the width of the film keeps increasing. This is done to investigate the effect of the thickness at the moment of reaching the buckling threshold and afterwards. The growth rates are relative to the reference configuration and the principal directions of volume growth (figure 3).

For each set of growth tensors, the two boundary conditions used correspond to a case in which the bottom of the system remains flat and a case in which the bottom boundary is given by *f*(*t*) = *r*(*t*)*x*(*x* − *w*_*o*_), with *r*(*t*) = *kt*, *k* = 0.2 for *t* < *τ* = 0.65 and *r*(*t*) = *kτ* otherwise, which emulates bulging due to turgor pressure. These results are also shown in figure 4 and are included to illustrate several important concepts and effects.

‘Time’ has a specific meaning in the model. The solutions of (2.2) are obtained iteratively as elastic responses to updates of *g*_*f*,*k*_ and *r*. Therefore, *t* controls relative changes due to growth for two or more internal parameters simultaneously. However, these solutions correspond to stationary configurations ignoring any transient dynamics, and in this respect time can be regarded as an internal clock-type parameter.

Regardless of the thickness growth, if the base of the substrate is deformed by increasing *r* then more expansive growth *g*_1,*f*_ is necessary to reach the stress buckling threshold. Here, the deformation of the base induces a strain across the entire domain, particularly in the cuticle film layer. Such stress needs to be compensated for in order to reach the buckling threshold. An immediate consequence of the delay is that, if the thickness of the film increases during that period, then the pattern wavelength is expected to be larger in accordance with (1.1). For the case in which the thickness stops growing before reaching the threshold, the delay also occurs but the change in wavelength is far less dramatic, the small difference is due to the extra length induced by the curved boundary, which allows the accommodation of an extra bump. Figure 4 shows the resulting patterns for each of the growth tensors and boundary conditions at the onset of the flat systems $t=t_{f}^{*}$, at the onset of the curved cases $t=t_{c}^{*}$ and at the end of the computation *t* = 1.

Once the pattern has been triggered, the effect of further film expansion in the first principal direction is to increase the amplitude of the pattern without changing the wave number. The morphology remains stable at least until a secondary instability is triggered [24,27–29].

The secondary instabilities for the planar setting discussed so far consist of period foldings (doubling/tripling) which are triggered simultaneously once a second buckling threshold is reached. Before discussing more on this, we discuss the result of investigating the breaking of the translation invariance which leads to stress thresholds which are reached at different instants of the layer development.

**Figure 5.**
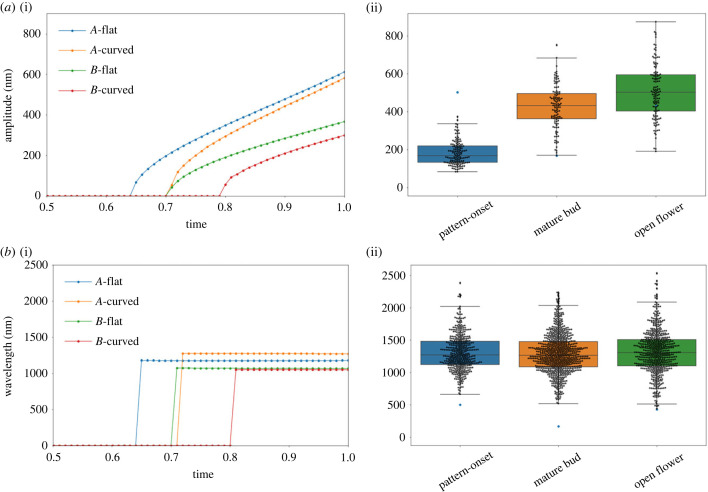
Amplitude and wavelength changes during *H. trionum* petal development and the effect of curved base boundaries. (*a*) The surface instability of the layer is triggered once a critical growth threshold is reached. The effect of a continuing film expansion after the pattern is triggered is the increase of the amplitudes. The pattern takes longer to fully populate the surface on curved substrates compared with the flat case. This implies that different curvature will lead to different amplitudes, which contributes to the variance observed in the data shown. (*b*) The wavelength across the surface as the amplitude increases remains constant in flat domains, which is not true in curved substrates. The average wavelength across the domains for curved and flat domains is also a non-zero difference, between all the cases considered here. (*a*)(i) Averaged striation amplitudes for the film expansion scenarios in figure 4. (ii) Box-plots showing measurements of striation amplitude in *H. trionum*. The curved boundary cases reach the threshold with a delay relative to the flat boundaries, which means lower amplitudes relative to their flat counterparts. (*b*)(i) Average striation wavelength for the film expansion scenarios in figure 4. (ii) Wavelength measurements from *H. trionum* at several stages of development. The box plots show an almost constant median. From the simple results of the simulations, we can see that reaching the threshold at different instances of development and with different thicknesses induces variance in the local wavelength which might also explain the variance observed in the data.

Figure 5 shows the amplitudes and wavelengths of the solutions shown in figure 4 alongside measurements of the same properties from *H. trionum* petals at several stages of development. From the figure, we see that for both sets of growth tensors, we obtain amplitudes and wavelengths in good agreement with those of the flowers by setting the width of the domain *w*_*o*_ = 〈*w*〉_12_, where *w*_*o*_ is the initial width of the cell. However, some effects are of note. First, the buckling threshold in the case in which the film thickness does not stop growing is slightly lower than the case in which the thickness expansion is stopped. This is expected as, in general, points in the deformed, current state are linear combinations of the local principal directions, and therefore contain contributions of both growth laws (figure 3). Second, in accordance with equation (1.1), the case in which the threshold is reached with a thicker film selects a pattern with a larger average wavelength.

Comparison of the cases using the same growth tensors but with different boundary conditions, namely, flat substrate base and curved substrate base, reveals a delay in reaching the stress threshold to trigger the instability in curved substrates. This provides a good indicator of why the pattern is triggered in patches mostly on top of the junctions between cells, and as shown in figure 2*b*, where it is shown that the pattern populates flatter domains first then regions with larger local curvature.

### 2.4. Multiple cells and substrate expansion

In order to explore in more detail the effects of epidermal cells with slightly different widths and cuticle base deformations, we now present results using a boundary condition which emulates the turgor-induced curvature. We achieved this by dividing the bottom boundary of a reference configuration into three main segments of lengths $w_{o}^{l}$, $w_{o}^{m}$ and $w_{o}^{r}$ located between small clamped intervals. Each of the segments is subject to the conditions $f_{k}=r_{k}(t)(x-x_{0}^{k})(x-(x_{0}^{k}+w_{0}^{k}))$ where *k* ∈ {*r*, *m*, *l*} stand for right, middle and left, and $w_{0}^{r}<w_{0}^{l}<w_{0}^{m}$.

**Figure 6.**
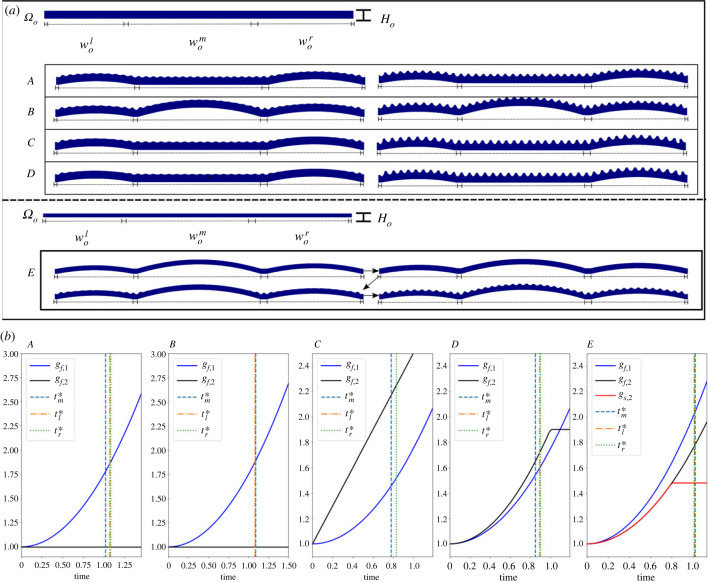
Multiple cells and non-uniform base deformations. (*a*) Solutions obtained from emulating three cells of slightly different length and growth rates as shown in (*b*). From this figure, we can see that the process of pattern formation is well described by the model. The pattern starts forming in regions above the cell junctions and in flat regions. The solutions (*A*–*D*) show the configuration in which the pattern has populated the region for the first time and the final configuration. The sequence in *E* corresponds to the case in which the substrate thickness is also allowed to increase, the pattern formation process, however, still follows the same steps. The average wavelength and amplitudes for these solutions are shown in figure 7. (*a*) Reference configurations and solutions for the growth scenarios shown below. The parameters used in *A*, *B*, *C*, *D*, *E* are *w*_*o*_ = 6.0, $w_{o}^{l}=1.48$, $w_{o}^{m}=2.4$, $w_{o}^{r}=1.84$ and *h*/*H*_*o*_ = 0.0066. *H*_*o*_ = 0.15 for *A*, *B*, *C*, *D* and *h*/*H*_*o*_ = 0.0066, allowing only film expansion in both principal directions. *H*_*o*_ = 0.07 for *E*, where we allow substrate expansion in the second principal direction. *R* = 20 and *ν* = 0.48. (*b*) Growth scenarios used to obtain the solutions above. The vertical lines indicate the time at which the threshold is reached in every cell.

Using the growth tensors provided in figure 6*b*, we emulate the pattern development over cells of different width and turgor-induced curvature. Solutions at the onset and fully formed patterns are shown in figure 6*a*, for the scenarios labelled *A*–*E*.

Cases *A* and *B* are subject to the same growth rates, allowing only film expansion in the first principal direction, with *r*_*m*_(*t*) = 0 for *A*. In *B*, every sub-domain is curved and given by the same rule as for the curved cases in figure 4. For *C* and *D*, the film expands in the two principal directions, keeping the middle domain flat.

In case *E*, we also consider growth of the substrate. Again, the buckling threshold is reached earlier in those sub-domains which remain flat (*A*, *C*, *D*) and the segments for which the boundary corresponds to the clamped junctions. A consequence of the delay in reaching the threshold is that the wavelength across the domain becomes less regular than for the flat case. This effect is partially geometrical and partially elastic. The geometrical effect is easy to understand if one considers a periodic flat curve described by (*x*, *h*(*x*)) and maps it into a curved convex segment given by **g** = (*x*, *g*(*x*)). The parametrization $u=g+h(x)\hat{N}$ achieves this, where $\hat{N}=(-t_{y},t_{x})$, *t*_*x*_ and *t*_*y*_ are **g**’s unit normal and the components of its tangent field, respectively.

If *h*(*x*) is for instance *A*cos (*kx*), we can map the values at *x*_*n*_ = (2*π*/*k*)*n* to points **u**_*n*_ = (*x*_*n*_ − *At*_*y*,*n*_, *g*_*n*_ + *At*_*x*,*n*_) and compute $\lambda_{n}^{2}=|u_{n+1}-u_{n}{|}^{2}$, which can be written as

2.5 $$\begin{array}{r} \lambda_{n}^{2}=\lambda^{2}+{\left(\Delta g_{n}\right)}^{2}+A^{2}(\Delta t_{x,n}^{2}+\Delta t_{y,n}^{2})+2A(\Delta t_{x,n}-\lambda \Delta t_{y,n}), \end{array}$$

with *λ* = *x*_*n*+1_ − *x*_*n*_ = 2*π*/*k*, Δ*g*_*n*_ = *g*_*n*+1_ − *g*_*n*_, and Δ*t*_*j*,*n*_ = *t*_*j*,*n*+1_ − *t*_*j*,*n*_.

The first two terms in the right-hand side of equation (2.5) are the hypotenuse of a triangle spanned by the difference in height in the concave profile and *h* evaluated at consecutive maxima. The last two terms make explicit that the larger the amplitude, the larger the separation between two consecutive peaks as these radiate away due to the concavity of *g*(*x*). This latter contribution is weaker as it only depends on differences in tangents evaluated at consecutive *x*_*n*_. This simple observation shows that the wavelengths across the concave curve are not constant, which explains in part the large variance observed in *H. trionum* petals (figure 5*b*). Another source of variance is set by mechanics, once the threshold is reached in a given sub-domain with a given wave number, then, the amplitudes start to increase asynchronously with respect to other sub-domains with a different local geometry, inducing relative differences in the wavelengths.

**Figure 7.**
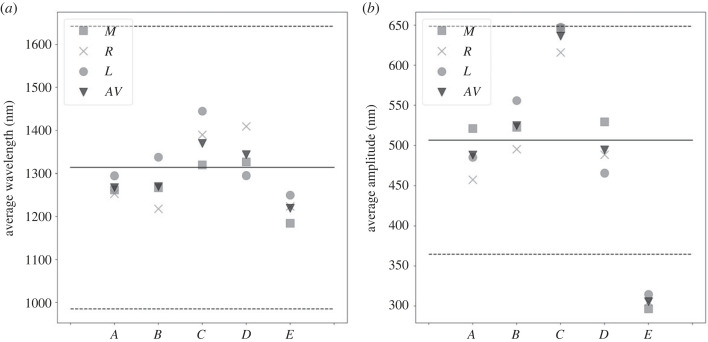
Average wavelength (*a*) and average amplitude (*b*) for each scenario in figure 6*a*. These are scaled using the same factor as for the single-cell case. The mean values and the standard deviation obtained from measurements of those quantities in *H. trionum* are also shown (solid and dashed lines). The labels *M*, *R*, *L* stand for middle, right and left cuticles whereas *AV* is the average value across the domain.

Average values of each sub-domain and across the full domain of amplitudes and wavelengths are shown in figure 7 for each case shown in figure 6. For *A* and *B*, where the only difference is the boundary condition of the middle cell, the average wavelength across the full domain is very similar, although due to the delay in reaching the threshold across the whole domain it takes more time to reach a similar set of amplitude values. For the growth scenarios *C* and *D*, the film is allowed to expand anisotropically in the two principal directions. As expected the effect of reaching the buckling threshold with a larger thickness leads to an increase in the pattern average wavelengths.

In all four cases, the non-uniform turgor boundary conditions induce differences in the required film expansion to reach the threshold locally. If the film also increases its thickness, there is a contribution which might reduce the expansion required to buckle. In general, however, curved boundaries always require larger in-plane expansion with respect to the flat case to buckle. The non-homogeneous boundaries induce wavelengths which exhibit variations across each sub-domain but on average are still qualitatively described by a scaling law like equation (1.1).

Case *E* corresponds to a case in which the substrate increases its thickness by setting *g*_*s*,2_ = *g*_*f*,2_ for *t* ∈ (0, 0.8) after which the deposition process stops. The film continues its expansion in both principal directions as indicated in the figure. The pattern formation follows a similar process as those described in the previous cases. This suggests that, to an extent, the thickness expansion of the substrate in the process of buckling does not play a relevant role on its own. To investigate the role of substrate growth further, we briefly present the results of studying the system in a full three-dimensional setting.

### 2.5. Volumetric case

**Figure 8.**
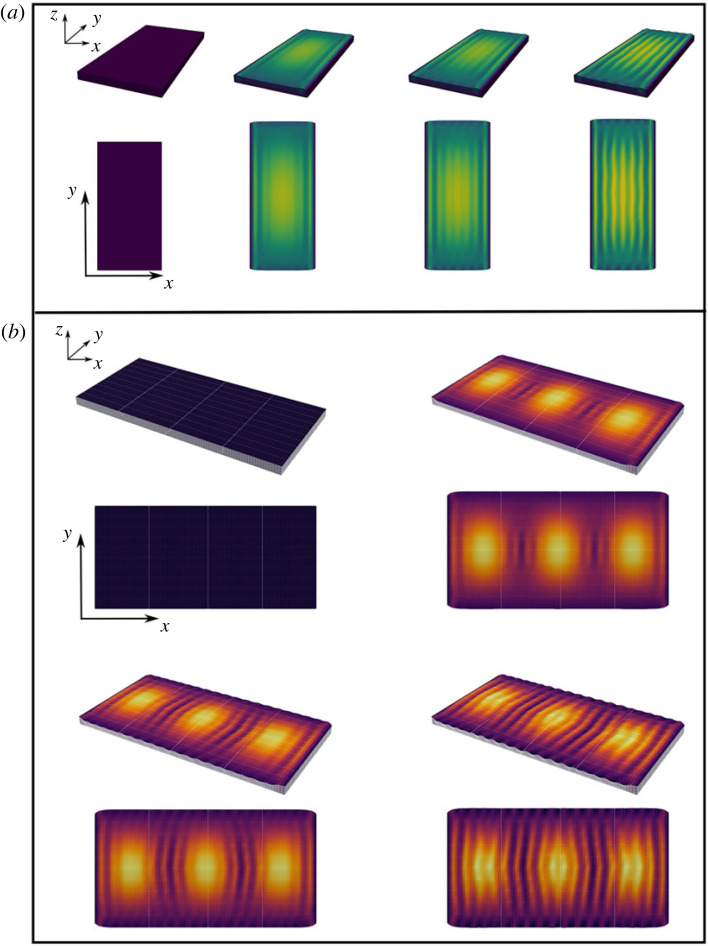
Volumetric growth. (*a*) Full volumetric growth, except for the film thickness. The base of the volume emulates deformation due to turgor pressure. The initial asymmetry and elongation overcome the growth-induced stress in the elongation direction. (*b*) Solutions obtained using a bottom surface boundary condition which emulates three cells. The pattern is triggered in the flatter regions and the cell junctions, which adds some disorder to the final configuration.

First, we present results of considering a reference configuration $\Omega_{o}=[0,w_{o}]\times [0,l_{o}]\times [0,H_{o}]$ with *l*_*o*_ = *w*_*o*_ = *H*_*o*_/10, *H_o_* = 1 and *h* = *H_o_*/41 as shown in figure 8*a*. The solutions are obtained imposing the boundary condition (*x*, *ty*/*l*_*o*_, 0.1*t* sin(*πx*/*w*_*o*_)sin(*πy*/*l*_*o*_)) on the base boundary surface to emulate turgor deformation. This boundary is stretched alongside the lateral surfaces in one preferred direction, emulating the expansion of the underlying cell. This is done by mapping the surfaces (*x*, *y*, 0) into (*x*, *ky*, 0), (0, *y*, *z*) into (0, *ky*, *z*) and (*w*_*o*_, *y*, *z*) into (*w*_*o*_, *ky*, *z*). The surfaces at the top and bottom of the length direction are free. The growth tensor entries are updated as: *g*_*f*,1_ = *g*_*f*,2_ = *g*_*s*,3_ = 1 + *t*. The amplitude of the turgor conditions is given by 0.1*t* and *g*_1,*s*_ = *g*_2,*s*_ are also set to be 1 + *t* but only for *t* < 0.2.

The turgor deformation effect is the same as for the planar simulations, namely a delay in reaching the buckling threshold in those points with larger bulging. The asymmetric initial configuration and the subsequent elongation of the domain in the length direction suffices to develop the oriented pattern, despite the in-plane isotropic growth. A slight undulation due to the growth-induced stress in the elongation direction can be seen; however, it becomes less noticeable as the volume keeps elongating. This is also observed in the flowers during development; in the mature bud stage, we note slight undulations in the striations (figure 1*j*) that disappear in the open flower once the cell length increases further (figure 1*k*).

The addition of volume into the bulk of the substrate and the film are necessary to describe the observations of the cuticle as the petal develops, as no significant decrease in cuticle thickness is observed across the maturing tissue. However, we note that this finding is for the average cuticle thickness. Our current sample preparation methods and measurement methods did not allow measurements of spatially local changes in thickness, which may provide further insight in future studies.

Finally, figure 8*b* shows a similar numerical experiment to the case of three cells discussed earlier. In this case, a region $\Omega_{o}=[0,l_{o}]\times [0,l_{o}]\times [0,H_{o}]$ is used as reference configuration and the bottom boundary given by *u*(*x*, *y*, *t*) = *r*(*t*)sin^2^(*k*_1_*x*)sin^2^(*k*_2_*y*), with *k*_1_ = 3*π*/*w*_*o*_ and *k*_2_ = *π*/*w*_*o*_. This emulates three underlying cell bulges in the domain. The remaining boundary conditions are the same as in the previous case.

The sequence shows results analogous to the three cells planar results. Specifically, the pattern is initiated at the regions which are flatter and the junctional regions. However, this example illustrates that the asynchronous pattern onset leads to configurations which are less ordered than the planar case. Again the source of in-plane stress is due to the lateral boundary conditions. The period and functions over which the turgor amplitude increases and these in-plane substrates grow are given by *r*(*t*) = *at*, *g*_1,*s*_ = *g*_2,*s*_ = *bt* + 1, for *t* < *τ* and *t* < 2*τ*, respectively (*τ* = 0.12), whereas the film changing entries are *g*_1,*f*_ = *g*_2,*f*_ = 1 + *bt*. The growth rate of the domain boundaries in the length direction is *y*(*t*) = 0.5 * *bt*. All these with *a* = 0.2, *b* = 1.5 and *t* ∈ [0.0, 0.25].

The figure shows that the pattern starts forming in the region between the bulges where the maxima of *u* are located (sin^2^(*k*_2_*y*) = 1) and in the vicinities of these peaks, then the pattern populates those regions with flatter support, and finally it populates the domes. This asynchronous pattern triggering coincides with our observations of pattern initiation in *H. trionum*. The presence of three domes also generates a pattern which appears more ‘organic’ than the previously discussed single-cell case. In the real system, these effects are of course enhanced by different curvatures, different cell sizes and divisions of the underlying cells as well as irregularities in the cuticle material properties and geometric features.

## 3. Discussion and conclusion

By tracking the development of the cuticular striation pattern on petals of *H. trionum*, we have provided a clear picture of how such patterns are triggered. As the cells elongate in one preferred direction and the area of the petal increases, the in-plane area of the surface cells increases, as does the thickness of the top layer of the cuticle, the cuticle film. The increasing area of the average cell surface leads us to postulate that compressive forces arise from expansion of the cuticle, and that cuticle growth is thus the main mechanism behind the compressive stress responsible for the surface buckling. This is compatible with previous suggestions of ‘cuticle overproduction’ as the driving force and with observations from other systems of patterned cuticle [2], which may be periodically oriented in a preferred direction, radially oriented wrinkles, or less coherent and disordered configurations. Equipped with this assumption, we then present a class of models which incorporate volume expansion into mechanical responses of a bilayer system.

In our models, cuticle production is interpreted as a local volume expansion of the layers, which results in in-plane stress. If the stress produced reaches a threshold then a pattern is triggered (critical growth condition), the threshold is determined by the stiffness mismatch ratio *R* > 1 condition.

A first thing to note is that buckling instabilities are triggered only after in-plane stress in a given direction exceeds a threshold value. In our case, this translates into the critical growth condition. If the expansion of the layer does not occur, then the system under consideration will not develop a buckled surface pattern. Also, even if cuticle is continuously produced, if the relative stiffness of the two layers is close to *R* ≃ 1 then a stable pattern will not form.

Therefore, a mono-layer system or a bilayer far from the threshold are adequate models for cuticles which retain a flat surface. Our previous work has demonstrated that the abaxial epidermal surface of the *H. trionum* petal does not buckle under mechanical stress, and we hypothesize that this is due to altered mechanical and chemical properties, most likely a stiffness ratio between layers that is close to 1 [11].

The scenarios explored here generate buckled morphologies following developmental paths resembling those of the *H. trionum* petal (figures 2, 5, 7 and 8). Specifically, by imposing non-homogeneous boundary sub-domains, patterns are triggered locally in those regions with smaller curvatures before populating the full domain (arrows in figure 2*c*).

Once pattern formation is initiated, the effect of continuing cuticle expansion is an increase in the pattern amplitudes, while the wavelength remains constant on average. However, local differences in curvature introduce differences in the amount of in-plane growth required to reach the threshold. This effect, in turn, causes the pattern to be triggered asynchronously, occurring first in flatter regions and at cell junctions. This explains why the pattern is observed to develop in patchy domains, before covering the fully ridged petal sub-domain in *H. trionum*. The delay in reaching the buckling threshold has been numerically and analytically studied in [30] for systems with a curvature field present in the reference configuration. The asynchronous onset of the pattern and variations in the curvature over each cell induce variability in the wavelength values. We have provided a simple expression that accounts for the variability observed in the data obtained as a result of purely geometrical considerations (equation (2.5)), without appealing to differences in growth rates and/or spatial variations of material properties. The asynchronous triggering of the pattern is a consequence of the broken translation invariance due to the bulged boundary conditions. In the volumetric cases presented here, we emulate the cell elongation by imposing boundary conditions that stretch the material layers as well as increase their volume. This breaks another symmetry as the in-plane stress perpendicular to the elongation reaches the wrinkling threshold before the components in the direction parallel to it. Moreover, as explored in [31], this mechanism is universal to generate aligned patterns even if the dynamics under consideration presents an entirely different pattern. In fact, if the layers grow without allowing the domain to grow, the surface will develop a pattern dictated by the in-plane aspect ratio of the domain and the growth rates, ranging from parallel wrinkles, to zig-zag patterns to chequerboard and hexagonal patterns. The elongation has an effect of smoothing the undulations in the principal elongation direction, thus making the secondary instability threshold more difficult to achieve. This is an effect of the three-dimensional nature of the problem, in contrast to the two-dimensional version of the problem. In sum, for the case under consideration here, we argue that the secondary instability does not play a relevant role except to induce ‘dislocations’ of the ridges.

The results presented here are successful in describing the formation of the patterns present in *H. trionum* as a passive response to growth of bilayers constrained by boundaries, provided that *R* > 1. However, how these two conditions are met in some petal domains and not in others is still an open question, which requires a better understanding of the processes involved in cuticle formation, cell wall formation and their material properties [10,26,32].

Indeed, while our current model considers the petal cuticle as a passive biolayer system subject to internal forces due to growth and external forces due to cell elongation, it is clear that the living cells which make up the petal are themselves engaged in extensive communication and signalling, and that the dynamic interactions between them not only produce the material properties that are our focus here but might also act to generate conditions different from those expected when considering cells in isolation. A fuller analysis of this system would include analysis of the chemistry and material properties of individual cells, an analysis of the transcriptomes and proteomes of those cells, a consideration of intercellular signalling, and a tissue-level analysis of the resulting material properties. It is often assumed that new polymer chains are added exterior to the cell wall independently of the cell elongation process [33] as the result of active transport processes. However, it is pertinent to ask whether cell expansion plays a role in the fulfilment (or not) of the condition *R* > 1, as cell elongation requires regulated dynamic cell wall softening. How the two layers (film and substrate) differentiate and how cuticle production is coupled with elongation [34,35] are questions which require an understanding of the regulation of cuticle development.

The morphoelastic approach taken here does not take into account any viscoelastic effects, which play an important role in the development of certain plant aerial parts. For instance, other studies [8,9,36] have shown that tomato cuticles should be described as viscoelastic materials. Hyperelastic materials, such as the neo-Hookean material used here, are an excellent proxy for investigation of viscoelastic systems, provided the timescales of the material stress relaxation are slow with respect to the stresses acting upon the material [37].

We note that the model described in this paper remains theoretical, providing a framework for experimental testing. Although our model development was informed by analysis of cell size, shape and growth, and by characterization of cuticle thickness and layering, it remains the case that data on stiffness of the bilayer materials might support or alternatively rebut our hypothesis. In addition, even if measurements of all relevant properties in the *H. trionum* system were to support our model, it will still be necessary to test it experimentally in future studies by perturbing the key parameters and observing the effects on pattern formation.

The present work focuses on the primary instability as observed in *H. trionum*, and its role as the cause of structural colour in the petal. However, the present class of models can be used to study the appearance of further instabilities which might be present over different systems, as shown in [12]. The findings in our paper contribute to the broader literature on buckling instabilities in multi-layered systems, which have been used to study periodic mechanical patterns in epithelial layers [38,39] and organ development [40–42]. Our work represents a comprehensive account of the mechanical ingredients for nano-scale pattern formation in the cuticle of petal epidermal cells, and provides a theoretical framework within which cuticular patterning can be explained by mechanical forces acting at the tissue level.

## 4. Material and methods

### 4.1. *Hibiscus trionum* geometric measurements

— *Plant growth conditions*. *H. trionum* seeds were obtained from Cambridge University Botanic Garden. Plants were grown in a greenhouse with a controlled temperature of 21°C, and a 16 h daylight regime. Plants were grown using Levington’s M3 compost.
— *Microscopy*. Cell measurements were performed using a stereo microscope M205 FA-Leica. Film thickness was calculated using Cryo SEM fractures taken with a Zeiss-Quorum cryoSEM in the Sainsbury Laboratory Microscopy Core Facility with 3 nm platinum coating. We measured the wavelength using a VHX-5000 Keyence with a VH-Z500R/W/T objective and a 2000× magnification.

### 4.2. Numerical solutions

To solve equation (2.2), we implemented a Lagrangian description on a finite-element scheme using the FEniCS framework [43], with dolfin v. 2019.1.0. The problem is solved in variational form by specifying the weak form

4.1 $$\int_{\Omega =\Omega_{f}\cup \Omega_{s}}P\;:\;\nabla v\;dV=\int_{\Omega_{f}}P_{f}\;:\;\nabla v\;dV+\int_{\Omega_{s}}P_{s}\;:\;\nabla v\;dV=0,$$

where P is obtained using the strain energy densities of a compressible neo-Hookean material model

4.2 $$\Psi_{\;j}=\frac{\mu_{j}}{2}(I_{1,j}-3)-\mu_{j}\ln J_{e,j}+\frac{\lambda_{j}}{2}\ln^{2}J_{e,j},$$

where *μ*_*j*_ = *E*_*j*_/2(1 + *ν*) and *λ*_*j*_ = *E*_*j*_*ν*/((1 + *ν*)(1 − 2*ν*)) are the Lamé material parameters expressed in terms of *E*_*j*_ and *ν*, which for materials with the same *ν* satisfy: *λ*_*f*_/*λ*_*s*_ = *μ*_*f*_/*μ*_*s*_ = *E*_*f*_/*E*_*s*_ = *R*.

The elastic deformation gradients $A_{j}=FG_{j}^{-1}$ and the Cauchy–Green tensors $C_{j}=A_{\;j}^{T}A_{j}$ allow us to compute, *I*_1,*j*_ and *J*_*e*,*j*_ and *J*_*g*,*j*_, the first and third principal invariants of $C_{j}$, $A_{j}$ and $G_{j}$, respectively. Alongside the second Piola–Kirchoff tensors

4.3 $$S_{j}=2\frac{d\Psi_{\;j}}{dI_{e,j}}I+J_{e,j}^{2}\frac{d\Psi_{e,j}}{dJ_{e,j}}C_{e,j}^{-1}$$

and

4.4 $$S_{j}=J_{g,j}G_{j}^{-1}S_{j}G_{j},$$

which gives the desired stress tensors

4.5 $$P_{j}=FS_{j}.$$

For the results presented here, we iteratively solve the system equation (4.1) in an interval *t* ∈ [0, *t*_*f*_] by updating the growth tensors at every time-step according to some growth laws, as described in the main text. In this respect, the time dependence is a quasi-static process as we compute stationary-only configurations after an increase in volume relative to the reference configuration $\Omega_{o}$ as sketched in figure 3.

## Data Availability

Full codes and documentation for the results presented here and some other wrinkling systems can be found in https://github.com/calugo/wrinkles.
